# Perceiving Musical Note Values Causes Spatial Shift of Attention in Musicians

**DOI:** 10.3390/vision1020016

**Published:** 2017-06-07

**Authors:** Valter Prpic

**Affiliations:** Division of Psychology, De Montfort University, The Gateway, Leicester LE1 9BH, UK; valter.prpic@dmu.ac.uk; Tel.: +44-7521121560

**Keywords:** SNARC, Att-SNARC, musical note value, spatial shift of attention

## Abstract

The Spatial-Numerical Association of Response Codes (SNARC) suggests the existence of an association between number magnitude and response position, with faster left-key responses to small numbers and faster right-key responses to large numbers. The attentional SNARC effect (Att-SNARC) suggests that perceiving numbers can also affect the allocation of spatial attention, causing a leftward (vs. rightward) target detection advantage after perceiving small (vs. large) numbers. Considering previous findings that revealed similar spatial association effects for both numbers and musical note values (i.e., the relative duration of notes), the aim of this study is to investigate whether presenting note values instead of numbers causes a spatial shift of attention in musicians. The results show an advantage in detecting a leftward (vs. rightward) target after perceiving small (vs. large) musical note values. The fact that musical note values cause a spatial shift of attention strongly suggests that musicians process numbers and note values in a similar manner.

## 1. Introduction

The SNARC (Spatial-Numerical Association of Response Codes) effect [1] is taken as a major evidence of the coupling between numbers and space. Indeed, Dehaene, Bossini, and Giraux [1] found that participants were faster in responding with a left (vs. right) key-press for small (vs. large) numbers during a parity judgment task. The authors suggested that this effect is due to a direct correspondence between the position of a number on a spatially oriented mental number line [2] and the response position in the external space. The SNARC has been consistently demonstrated over time, both using tasks where the number magnitude was relevant (e.g., magnitude comparison [3]) and irrelevant (e.g., orientation judgment [4]). The origins of the SNARC effect are, however, still unknown. Several studies [5,6] suggest that the effect is culturally acquired and is related to the direction of writing/reading. Conversely, other studies [7,8] suggest that mapping numerical magnitudes in a left-to-right fashion can be a universal cognitive strategy independent from cultural factors.

A study conducted by Fischer, Castel, Dodd, and Pratt [9] extended this phenomenon, suggesting that number magnitude does not only influence motor responses but also affects the allocation of spatial attention. Indeed, in two experiments the authors demonstrated that merely perceiving numbers causes a spatial shift in covert attention. Both experiments required unimanual responses to a visual target appearing either on the left or on the right side of the screen. Before target appearance, a single digit was presented at fixation for 300 ms. The results show an advantage in detecting a left target after perceiving small numbers (i.e., 1 or 2) and a right target after perceiving large numbers (i.e., 8 or 9). Authors concluded that small number magnitudes produce a leftward shift of attention, whereas large magnitudes produce a rightward shift of attention. This effect, commonly named the Attentional SNARC effect (Att-SNARC), was demonstrated to be automatic and to occur without involving saccadic eye-movement. Furthermore, this effect was particularly robust when a delay of 400 and 500 ms occurred between the number and the target appearance, while it tends to disappear at shorter or longer delays.

Among the first studies to replicate this phenomenon, Galfano, Rusconi, and Umiltà [10] and Ristic, Wright, and Kingstone [11] are worth mentioning. In particular, Ristic, Wright, and Kingstone [11] aimed at investigating whether numbers trigger rapid reflexive shift of spatial attention similarly to other central directional stimuli (e.g., gaze direction and arrows). Their results clearly show that the effect triggered by numbers is not reflexive but, rather, it is strongly susceptible to top-down control. Indeed, the Att-SNARC effect can be simply reversed by asking participants to imagine a reversed mental number line (with small numbers on the right and large numbers on the left) or numbers depicted on a clock face. Galfano, Rusconi, and Umiltà [10] found similar evidence, suggesting that the spatial shift of attention mediated by number magnitude is not obligatory. The fact that the Att-SNARC effect is flexible and mediated by top down factors should not be surprising, since the "classical" bimanual SNARC effect was also revealed to be mediated by task instructions [12], the position of items in working memory [13], and cultural factors [5,6]. 

A study by van Galen and Reitsma [14] found evidence of the Att-SNARC effect in 9-year-old children and in adults, while a bimanual SNARC effect was found also in 7 and 8-year-old children during a magnitude comparison task. This evidence suggests that younger children (7 and 8 years old) do not automatically activate semantic information about number magnitude. Furthermore, the Att-SNARC effect seems to be number specific [15], while the SNARC effect was previously found also for ordinal sequences, such as letters of the alphabet, months of the year, and days of the week [16,17], and for several non-numerical magnitudes, such as physical size, luminance, angle magnitude, and loudness [18,19,20,21]. Indeed, Dood, Van der Stigchel, Leghari, Fung, and Kingstone [15] successfully replicated the Att-SNARC effect with numbers, but failed to do so with letters, months, and days. 

Another study [22] replicated previous findings, revealing a significant Att-SNARC effect for numbers but not for letters. Furthermore, this study showed that, similarly to what happens with gaze direction and arrows, participants showed an inhibition of return (IOR) at longer delays between stimulus and target presentation. Indeed, at the longer delay (1650 ms), participants were faster to detect a target in the uncued visual field compared to the cued visual field. Again, this effect was present only for numbers and not for letters, further suggesting that the Att-SNARC effect is number specific. Although several studies successfully replicated the Att-SNARC effect for numbers, it is worth mentioning that other studies failed to do so (for a review see [23]). Currently there is a strong debate around the mixed outcome of the replication studies and more investigations are needed to identify the possible moderators and the underlying mechanisms of this effect. 

A growing number of studies investigated SNARC-like effects in psychomusicology. Most of these studies showed that music related stimuli are also closely linked with space (for a review see [24]). The first studies to investigate SNARC-like effects with musical stimuli focused on pitch height. Both Rusconi, Kwan, Giordano, Umiltà, and Butterworth [25] and Lidji, Kolinsky, Lochy, and Morais [26] showed that both musicians and non-musicians automatically associate high (vs. low) pitches with top (vs. bottom) responses, while only musicians associate high (vs. low) pitches with right (vs. left) responses. A more recent study that compared the performance of a group of piano and flute players [27] showed that specific music training can have an influence on the strength of this association. In particular, the pairing between high (vs. low) pitches and right (vs. left) location was strengthened in the group of piano players after performing their instruments. Furthermore, another recent study [28] revealed that, at least in non-musicians, the SNARC-like effect for pitch height is influenced by the brightness of the tone’s timbre. 

Temporal aspects of music have also been shown to elicit SNARC-like effects. In particular, the STEARC (Spatial-Temporal Association of Response Codes) effect revealed that time is represented from left-to-right along the horizontal axis [29]. In particular, the authors of this study found that left-side responses were faster for early onset timing, while right-side responses were faster for late onset timing. Similarly, also music tempo seems to elicit a similar effect, with faster left-key responses for slow beat sequences and faster right-key responses for fast beat sequences [30]. Furthermore, an extensive number of studies suggest that space and time are tightly linked, providing evidence of a mental time line (for a review see [31]). 

Although a growing amount of literature investigated SNARC-like effects for music related stimuli, little is known about the influence of these kinds of stimuli on spatial attention. One study [32] that addressed this specific topic investigated the influence of pitch height to the allocation of attention with an attentional cuing paradigm. The authors found that high and low tones induce attentional shifts to upper or lower locations, respectively. Similarly to the Att-SNARC effect for numbers, the effect of pitch height on spatial attention also seems to be quite flexible and susceptible to top-down control.

The aim of the current study is to replicate the Att-SNARC effect in a group of musicians by using musical note values instead of numbers. Musical note values can be considered the equivalent in music of numbers in mathematics. Indeed, note values are the symbolic representation of the relative duration of notes and have many similarities with digits. A previous study [33] showed a clear bimanual SNARC-like effect for musical note values both during magnitude relevant (note value comparison) and irrelevant (line orientation judgment) tasks. However, whether musical note values can act as attentional cues, orienting visuo-spatial attention to the left or right visual field depending on the note value still remains an open question. The hypothesis of this study is that, after perceiving small note values (eighth and sixteenth notes) musicians will be faster in detecting a target appearing in the left visual field, while, after perceiving large note values (whole and half notes), musicians will be faster in detecting a target in the right visual field. 

## 2. Results

Outlier RTs below 150 ms were removed from the data (2.1%), according to the previous literature [14]. The rest of the data were analyzed using a linear regression analysis as proposed by Fias, Brysbaert, Geypens, and D’Ydewalle [34] and were adopted in previous studies on the Att-SNARC effect [14]. The predictor variable was the musical note value, whereas the criterion variable was the difference between the reaction time (RT) in detecting the right and the left targets: dRT = RT(right target) − RT(left target). Positive dRTs indicate faster RT in detecting a left target, whereas negative dRTs indicate faster RT in detecting a right target. In a first step, for each participant the median RT was computed for each musical note value, separately for the left- and right-target. On the basis of these medians, dRT was computed by subtracting the median RT of left-target detection from the median RT of right-target detection. In the second step, a regression equation was computed for each participant with the musical note value as the predictor variable. In the third step, a one-sample t-test was performed to verify whether beta regression weights of the group deviated significantly from zero.

**Figure 1:**
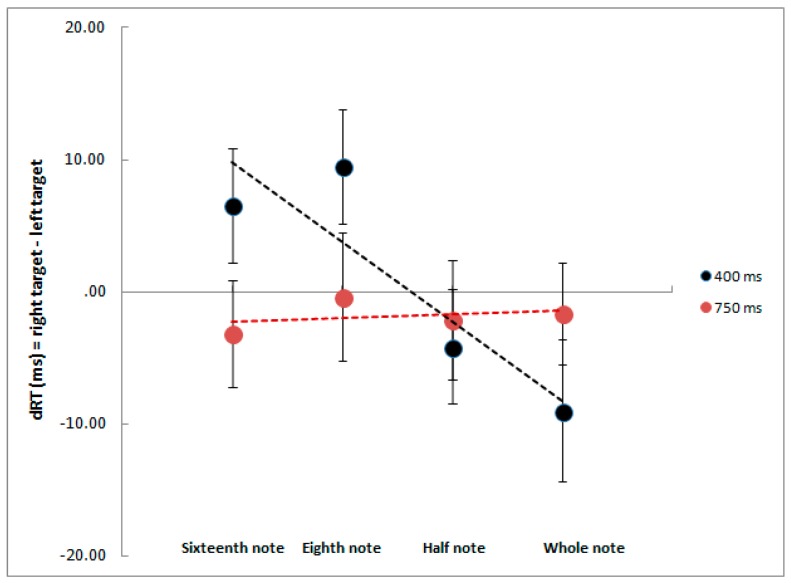
Mean differences of the median RT (response time) right target – RT left target as a function of musical note values with a 400 ms and 750 ms delay. Positive differences indicate faster left-target detection; negative differences indicate faster right-target detection. Error bars represent standard errors of the mean.

The analysis of dRT revealed that the regression slopes were significantly different from zero in the short delay condition (400 ms), *t* (26) = −3.02; *p* < 0.01, but not in the long delay condition (750 ms), *t* (26) = 0.68, *p* = 0.50. Therefore, as shown in Figure 1, there was a relative left target detection advantage after the presentation of small note values (i.e., eighth note and sixteenth note), and a relative right target detection advantage after the presentation of large note values (i.e., whole note and half note) in the short delay condition. Conversely, no target detection advantage was revealed in the long delay condition.

## 3. Discussion

Results show that musical note values (i.e., the relative duration of notes) can act as attentional cues, orienting visuo-spatial attention to the left or right visual field depending on the note value. Indeed, after perceiving small note values (eighth and sixteenth notes) participants were faster in detecting a target in the left visual field, while, after perceiving large note values (whole and half notes), they were faster in detecting a target in the right visual field. 

The fact that musical note values cause a spatial shift of attention strongly suggests that musicians process note values similarly to numbers. Previous studies [15,22] failed to replicate the Att-SNARC effect by using stimuli other than numbers (e.g., letters) and, therefore, authors concluded that the Att-SNARC is number specific. However, musical note values have much more in common with numbers than with letters of the alphabet, since both note values and numbers provide both magnitude and ordinal information, while letters can provide only ordinal information. Indeed, the lack of magnitude information is, thus, the most probable reason why letters failed to show the Att-SNARC effect. There are, however, very few studies that investigated the Att-SNARC effect by using magnitude related stimuli other than numbers. One of these, Luccio, Fumarola, Tamburini, and Agostini [35] showed that an Att-SNARC effect can also be elicited by non-symbolic numerical quantities (array of dots). Thus, magnitude information seems to cause a spatial shift of attention independently of its format. 

The reason why ordinal and magnitude information lead to different results in the SNARC and Att-SNARC effects is not fully understood. The association between ordinal stimuli (e.g., letters) and space seems to be task dependent, and indeed a clear SNARC-like effect for letters is usually found in tasks where the participants are forced to make an order-relevant decision (e.g., does C precede or follow M?) [15,16,17]. Conversely, the association between numbers and space is consistently revealed with different task demands and seems to be highly automatic, as shown by the Att-SNARC. This could be because integers have strict ranking properties that are defined by incremental relationships (e.g., 2 is always smaller than 3 and will always precede it in the ranking order), while this is not true for letters. Indeed, letters are used freely to compose words and there are no strict ordinal relationships between them. Therefore, the ordinal relationship between letters is arbitrary by nature and it became salient only if we require an order-relevant decision that forces us to process letters in alphabetical order. Dood, Van der Stigchel, Leghari, Fung, and Kingstone [15] also suggest that there might be an interaction between learning experience and neural structures that has developed for numbers. Furthermore, the authors suggest that this might develop for other ordinal sequences due to extensive experience. The current study supports this claim. Indeed, note values have many properties in common with numbers and they are highly overlearned stimuli for musicians. 

The Att-SNARC effect for musical note values was revealed in the short (400 ms) but not in the long (750 ms) delay condition, suggesting that this effect rapidly decays in time. One possible explanation for this is that music reading is usually a fast process that proceeds with a sustained pace. Attention is allocated to each note for a short amount of time and then reallocated to the next one. Indeed, 750 ms could actually be a very long interval for expert music readers and this could account for a rapid decay of the effect for musical note values. Even so, the time interval in which the effect was revealed seems partially in line with the ones of previous studies on the Att-SNARC effect. Indeed, whether Fischer, Castel, Dodd, and Pratt [9] found a robust Att-SNARC effect for the number magnitude at 400 ms, 500 ms, and 750 ms, a more recent replication [15] failed to show a significant effect in the longer delay condition (750 ms). 

Overall, the Att-SNARC effect for numbers and musical note values seems to appear with a similar timing, further suggesting that musicians process those two categories in a similar manner. However, although the many similarities with the Att-SNARC effect were originally revealed for numbers, the spatial shift of attention for musical note values is an independent phenomenon that is likely to affect the musicians’ population only. Furthermore, whether these two effects are supported by common or separate underlying mechanisms should be assessed by future research. 

The current study replicates and extends previous findings showing that musical note values are spatially coded [33]. Indeed, the current study shows that not only do note values influence motor responses, they also affect the allocation of spatial attention. Musical stimuli have been often used in SNARC-like tasks in order to investigate the relationship between space and music parameters (for a review see [24]). For instance, the temporal aspects of music have a strict link with the horizontal axis, shown by the left response advantages for early onset timing and slow tempo, and right response advantages for late onset timing and fast tempo [29,30]. Similarly, Rusconi, Kwan, Giordano, Umiltà, and Butterworth [25] and Lidji, Kolinsky, Lochy, and Morais [26] revealed that pitch height can be spatially coded both along the horizontal and vertical axes. Furthermore, a recent study [32] showed that sounds with different pitches can also induce attentional shifts, similarly to numbers and musical note values. The fact that numerical and musical stimuli similarly affect both spatial motor responses and the allocation of spatial attention suggests that a special connection exists between mathematics, music, and space. 

## 4. Materials and Methods

### 4.1. Participants 

Twenty-seven participants, aged from 19 to 34 (16 females; M = 26.4 years, SD = 4.8 years) took part in the study. They all attended a music school for at least 3 years (M = 6.2, SD = 2.6), thus they were able to read music notation. Eleven of the participants studied piano, six studied violin, eight studied guitar, and two studied flute. However, most of them reported to play more than one instrument. They were all right-handed and used to the left-to-right writing direction. All participants had normal or corrected-to-normal vision and were naive about the purpose and the hypothesis of the study. Informed consent was obtained from all participants prior to participation in the experiment, which was conducted in accordance with the ethical standards established by the Declaration of Helsinki. The present study was approved by the Research Ethics Committee of the University of Trieste in compliance with national legislation.

### 4.2. Apparatus and Stimuli 

The experiment was created and controlled by means of the E-Prime software, version 2.0. Stimuli were displayed on a 15.6-inch screen, with a 1280 × 800 pixels resolution. A five button Serial Response Box, connected to the computer by means of a USB port, was used for collecting responses.

The stimuli were four images depicting four musical note values (whole note, half note, eighth note, and sixteenth note). Whole and half notes represented relatively long duration notes, while eighth and sixteenth notes represented short duration notes. The notes were depicted in the middle of the image against a dim yellow background. No musical staff or other cues appeared on the screen, thus only one isolated musical note was presented at a time.

### 4.3. Procedure 

**Figure 2:**
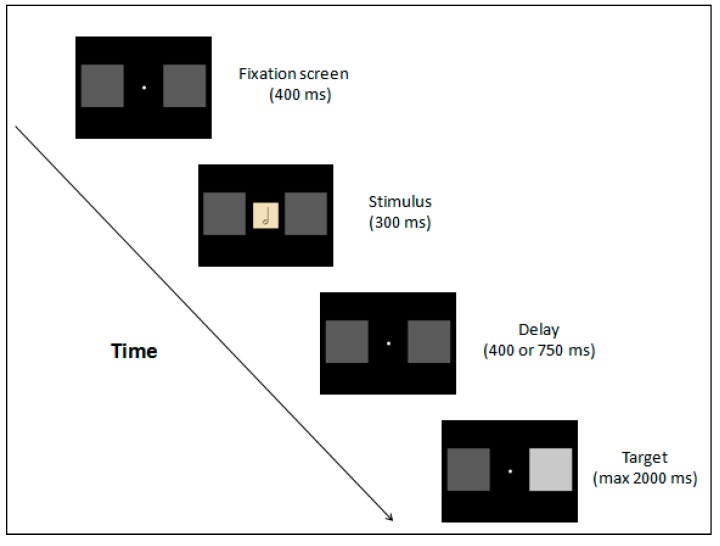
Task sequence. In this example, the stimulus was the half note and the target appeared on the right visual field.

The experimental procedure consisted of a detection task adapted from Fischer et al. [9] and van Galen and Reitsma [14] (see Figure 2 for the task sequence). The main difference with the previous studies was that instead of digits, musical note values were presented. The experiment took place in a quiet and dimly light room. Participants were positioned in front of the computer’s screen with a viewing distance of approximately 60 cm. The midlines of the screen and the response box were aligned with the midline of the participant’s body. Participants were instructed to move as little as possible.

Each trial started with a white fixation point displayed for 400 ms at the centre of the screen between two sided dark grey boxes. Thereafter, an image depicting one of the musical note values appeared for 300 ms at fixation. After a variable delay (400 ms and 750 ms), one of the two dark grey squares was replaced by a lighter grey square, that disappeared in 2000 ms or after participants responded. The reason for a variable delay was to encourage participants to wait for the target to appear, thereby avoiding time guessing. Both delays were within the range where the Att-SNARC effect was previously found [9,15]. Participants responded at the appearance of the light gray square target by pressing the central key on the response box. The images depicting musical note values were presented as a prime and no instructions related to the stimuli were given to the participants in order to perform the task. The inter-trial interval (ITI) was 1500 ms. 

The experiment was divided into two sessions. In the first one, participants were asked to press the central key with the right index finger at the target appearance, while, in the second one, participants pressed the central key with the left index finger. Speed was stressed in the instructions by asking participants to respond as soon as they detected the target. The order of the sessions was counterbalanced among participants. Before each session, participants completed 8 practice trials. A session consisted of a total of 80 trials where each musical note value was presented 20 times in random order. Participants were allowed to take a short break between the two sessions, otherwise they continued with the experiment.
